# Insight into the Pathology of a COL1A1 Signal Peptide Heterozygous Mutation Leading to Severe Osteogenesis Imperfecta

**DOI:** 10.1007/s00223-017-0359-z

**Published:** 2017-11-03

**Authors:** U. Lindert, M. Gnoli, M. Maioli, M.F. Bedeschi, L. Sangiorgi, M. Rohrbach, C. Giunta

**Affiliations:** 1 Connective Tissue Unit, Division of Metabolism and Children’s Research Center, University Children’s Hospital, Steinwiesstrasse 75, 8032 Zurich, Switzerland; 2 Department of Medical Genetics and Skeletal Rare Diseases, Rizzoli Orthopaedic Institute, Bologna, Italy; 3 Medical Genetics Unit, Fondazione IRCCS Ca’Granda Ospedale Maggiore Policlinico, Milano, Italy

**Keywords:** Osteogenesis imperfecta, Bone, Collagen, COL1A1, Signal peptide mutation, Signal peptide cleavage site

## Abstract

Osteogenesis imperfecta or “brittle bone disease” is a congenital disorder of connective tissue causing the bone to break easily. Around 85–90% of cases are due to autosomal dominant mutations in the genes encoding type I collagen, the major organic component of bone. Genotype–phenotype correlations have shown that quantitative defects of collagen type I lead to mild OI, whereas structural defects show a wide clinical range from mild to perinatal lethal. This may partially be explained by the type of amino acid substitution and the relative location in the domain structure. To fully understand the variability of the clinical manifestation and the underlying pathomechanisms, further investigations are required. Here we provide the first biochemical characterization of a mutation at the signal peptide cleavage site of COL1A1, a domain not yet characterized. By steady-state analysis, we observed reduced production of collagen type I. Furthermore, by pulse-chase analysis we detected delayed secretion and partial intracellular retention of collagen I. In the cellular fraction, the electrophoretic migration was abnormal; however, secreted type I collagen showed a normal migration pattern. The intracellular retention of collagen I was confirmed by immunofluorescent staining. Moreover, transmission electron microscopy of cultured fibroblasts revealed enlargement of ER cisternae. These results further support the hypothesis that mechanisms interfering with ER integrity play an important role in the pathology of severe OI.

## Introduction

Osteogenesis imperfecta (OI; MIM 166,200, 1,666,210, 259,420, and 166,220) is a heterogeneous heritable disorder of bone matrix formation and remodeling. The cardinal manifestations are bone fragility and deformity due to hypermineralization of the bone matrix resulting in stiffer bone tissue which is more prone to fractures [1–3]. Based on clinical and radiological findings, individuals with OI are classified according to Sillence as mild type I, perinatal lethal type II, severe type III, or moderately deforming type IV [4]. Around 85–90% of cases with OI are caused by mutations in the genes *COL1A1* (MIM 120,150) or *COL1A2* (MIM 120,160), encoding type I collagen [5]. Procollagen peptide chains in the rough endoplasmic reticulum (rER) undergo a series of modifications, such as hydroxylation and glycosylation, and assemble into a triple helix consisting of two α1(I) chains and one α2(I) chain, respectively. Glycine is required at every third position in the helical part since it is the only amino acid small enough to face into the inner side of the triple helix [6, 7]. After secretion into the extracellular space and removal of the N- and C-propeptides, the so-formed insoluble mature collagen I molecules can assemble into fibrillar structures.

Genotype–phenotype correlations have shown that *COL1A1* mutations leading to quantitative alterations of the transcript, in particular to haploinsufficiency, are associated with mild OI type I, whereas mutations leading to structural defects result in a wide clinical spectrum from mild to lethal forms. Glycine substitutions in the triple helical part account for the majority of these mutations and are associated with overmodification of the triple helix due to delayed folding. Starting from 2006, several non-collagen genes causing OI have been identified and shown to be involved in collagen I biosynthesis, bone mineralization, and osteoblast development (reviewed in [8]). Unfortunately, the pathophysiological understanding could not keep pace with the expanding insight into the genetic heterogeneity of OI. Therefore, it remains unclear whether common pathological mechanisms exist and whether these are mainly determined by collagen deficiency, cellular malfunction, or structural defects in the extracellular matrix.

**Fig. 1:**
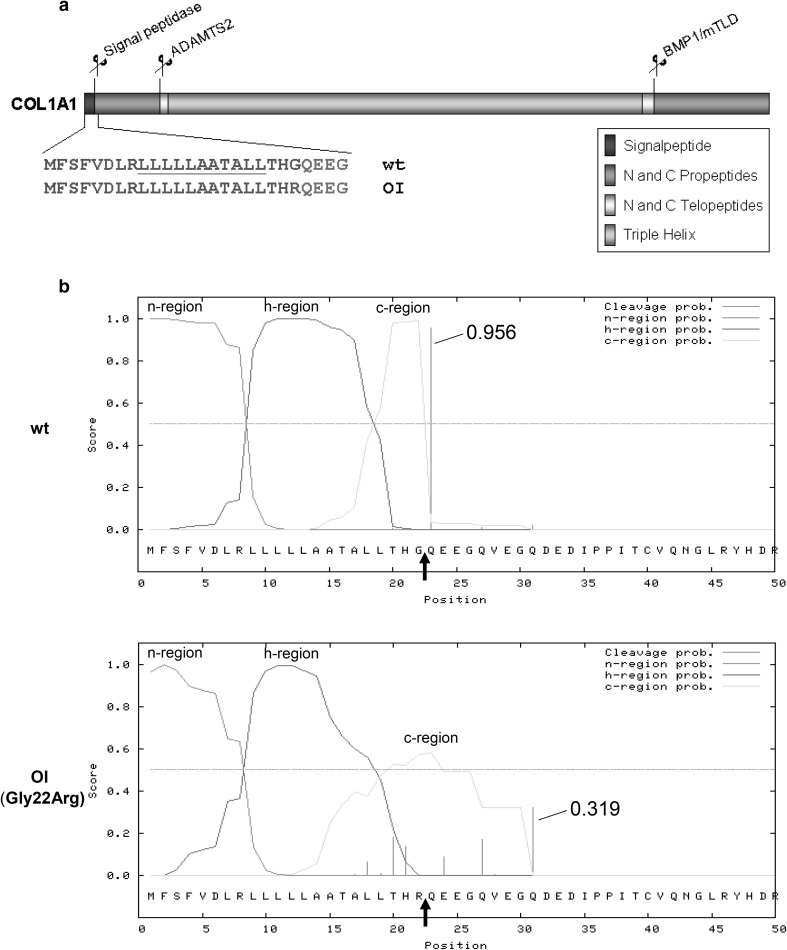
**a** Domain structure of COL1A1 protein with p.Gly22Arg substitution in the signal peptide highlighted in blue. **b** Prediction of the cleavage site probability of the wt *COL1A1* sequence and the p.Gly22Arg substitution using the SignalP algorithm. The algorithm predicts a strong reduction of the cleavage site probability (from 0.956 to 0.319) for the p.Gly22Arg substitution. The predicted cleavage site for the Gly22Arg substitution with the highest probability is at a different position (between Gly30 and Gln31). A black arrow indicates the position of the wt signal peptide cleavage site; n-region: N-terminal region; h-region: hydrophobic region; c-region: cleavage region (Color figure online)

Here we provide novel insight into the pathophysiology of OI by investigating a rare disease causing variant localized in the signal peptide of *COL1A1* [9], a hitherto functionally uncharacterized domain (Fig. 1a).

A heterozygous de novo p.Gly22Arg mutation at the − 1 position of the signal peptide cleavage site was identified in a fetus with severe OI, aborted at the 22nd week of pregnancy (patient ID: AN_000079 in the osteogenesis imperfecta variant database https://oi.gene.le.ac.uk/). The same mutation has been reported previously in a patient with OI type II [10]. Recently the p.Gly22-Gln23del mutation (deletion of the − 1 and + 1 positions of the signal peptide) has been identified in a patient with OI type III [11]. However, both cases were identified by epidemiological studies and thus no biochemical characterization was performed. Signal peptides contain a hydrophobic core, which allows the targeting of the protein to the ER upon translation, and a cleavage site which is required to release the protein from the membrane pore (translocon). Efficient cleavage by the signal peptidase requires small amino acids (Ala, Gly, Ser) at the − 1 position of the cleavage site [12]. To gain insight into the pathomechanism of this form of OI, we set out to analyze collagen biosynthesis and secretion, as well as the cellular ultrastructure.

## Methods and Results

### Genetic Analysis

All coding exons and flanking exon–intron junctions of *COL1A1* and *COL1A2* were Sanger sequenced using BigDye Terminator version 3.1 cycle sequencing kit and an ABI Prism 3100 automated DNA sequencer (Applied Biosystems, Foster City, CA).

### Cleavage Site Prediction

In silico analysis of the signal sequence was done by submitting the first 50 amino acids of the COL1A1 sequence to the SignalP 4.1 server at http://www.cbs.dtu.dk/services/SignalP/ [13].

A strong reduction of the cleavage probability (from 0.956 to 0.319) for the p.Gly22Arg substitution was predicted (Fig. 1b).

### Cell Culture and Collagen Analyses

Fibroblasts of the fetus were obtained from an umbilical cord biopsy by explant culture. Patient and control fibroblasts were grown under standard conditions using DMEM medium supplemented with 10% fetal calf serum, 100 units/ml of penicillin, 100 μg/ml of streptomycin, and 0.25 μg/ml of amphotericin B (Gibco). Fibroblasts of an unaffected individual in pediatric age were used as a control.

To assess possible effects on collagen biosynthesis and secretion, we performed collagen steady-state and pulse-chase analyses in cultured fibroblast as described [14] with minor changes: 4-h pulse labeling of collagen with (^3^H)-proline and (^3^H)-glycine was followed by chase with unlabeled amino acids for 5, 30 min, 2 and 24 h.

**Fig. 2:**
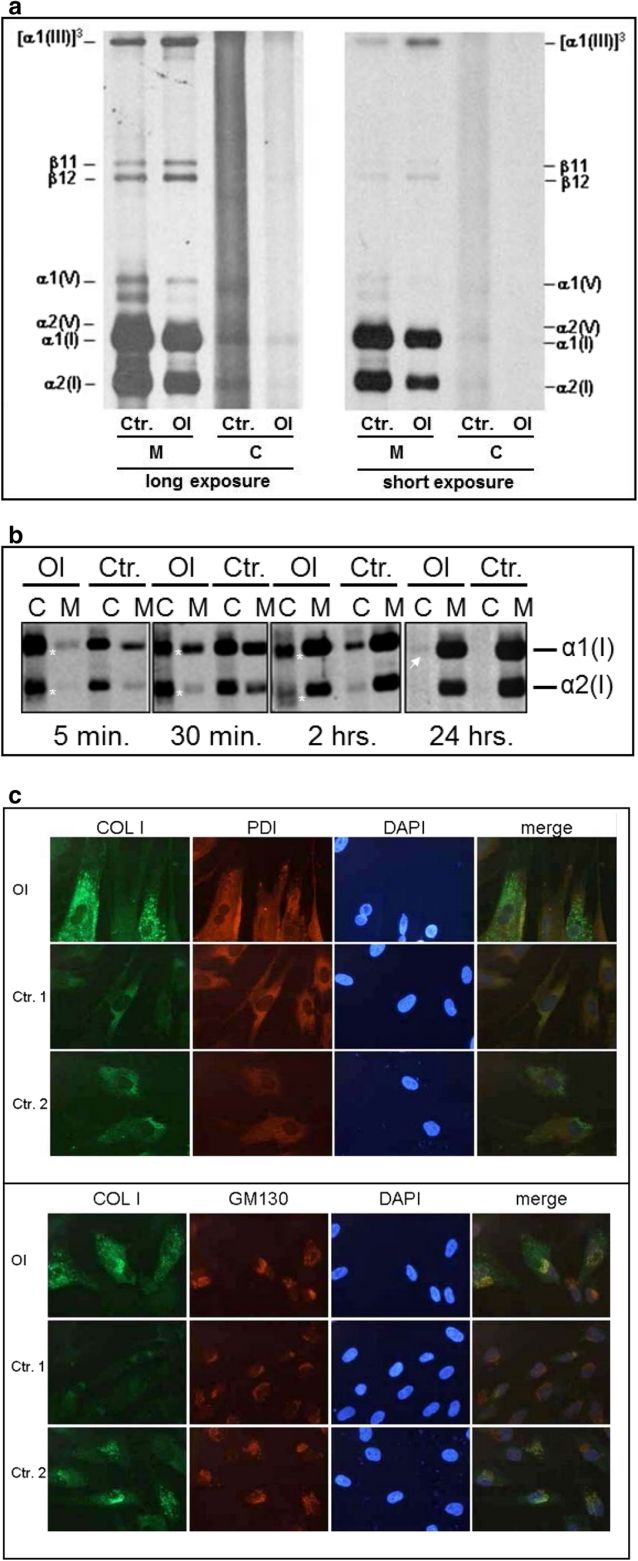
Collagen synthesis and secretion in fibroblasts cultures of the OI fetus (OI) and a control (Ctr.); **a** steady-state analysis showing decreased levels of type I collagen α1(I) and α2(I) relative to type III collagen [α1(III)]_3_ in the medium (M) of the OI fibroblasts suggesting that less collagen type I was produced and secreted by these cells; **b** pulse-chase analysis showing a decreased amount of type I collagen chains secreted from the cell layer (C) into the medium layer (M) in the OI fibroblasts compared to the control in the 5-min, 30-min, and 2-h chases. Also, bands with a broader migration pattern are visible in the cell layer (C) of the OI fibroblasts (white asterisk) and a small amount of collagen is still visible after 24-h chase in the cell layer of the OI sample (white arrowhead) but not the control; **c** Co-immunofluorescent staining of type I collagen (COL I) together with ER (PDI) or Golgi (GM130) markers. DAPI was used to stain nuclei and an overlay of COL I with either PDI or GM130 is given showing increased staining for type I collagen in the OI cells and only partial overlap with ER and Golgi markers

Steady-state analysis showed that the proportion of type I to type III collagen was reduced in the medium of the OI fibroblasts compared to the control (Fig. 2a).

Pulse-chase analysis showed that the secretion of the α1(I) and the α2(I) chains from the cells into the medium was slightly delayed in the OI fibroblasts (Fig. 2b). A small amount of type I collagen was retained in the cells even after 24 h (arrow, Fig. 2b). Furthermore, in the cell layer, but not in the medium of the OI cells, the α1(I) and α2(I) chains appeared as slightly broad and fuzzy bands, suggesting the presence of both overmodified as well as undermodified collagen chains in the 5-min, 30-min, and 2-h chases (Fig. 2b).

### Immunofluorescent Staining

To further investigate the intracellular distribution of collagen in the OI and control fibroblasts, co-immunofluorescent staining for type I collagen (COLI; mouse anti-collagen I Abcam ab6308; 1:100) and either the ER marker protein disulfide isomerase (PDI; rabbit anti-PDI Santa Cruz sc-20,132) or the Golgi marker GM130 (rabbit anti-GM130 Abcam ab52649; 1:100) was performed as described [14]. Secondary antibodies were goat anti-rabbit AF568 (ab175471) Abcam (1:500) and goat anti-mouse AF488 (A11017) Molecular Probes (1:500). Nuclei were stained with 4′,6-diamidino-2-phenylindole (DAPI). Type I collagen localized to the ER as well as to the Golgi in OI as well as control fibroblasts. Additionally, in the OI fibroblasts, a punctuate staining not overlapping with the ER or Golgi markers was observed (Fig. 2c).

### Transmission Electron Microscopy

To investigate whether the morphology of the ER was altered due to the putative retention of misfolded collagen, we performed transmission electron microscopy on cultured fibroblasts as described [14].

**Fig. 3:**
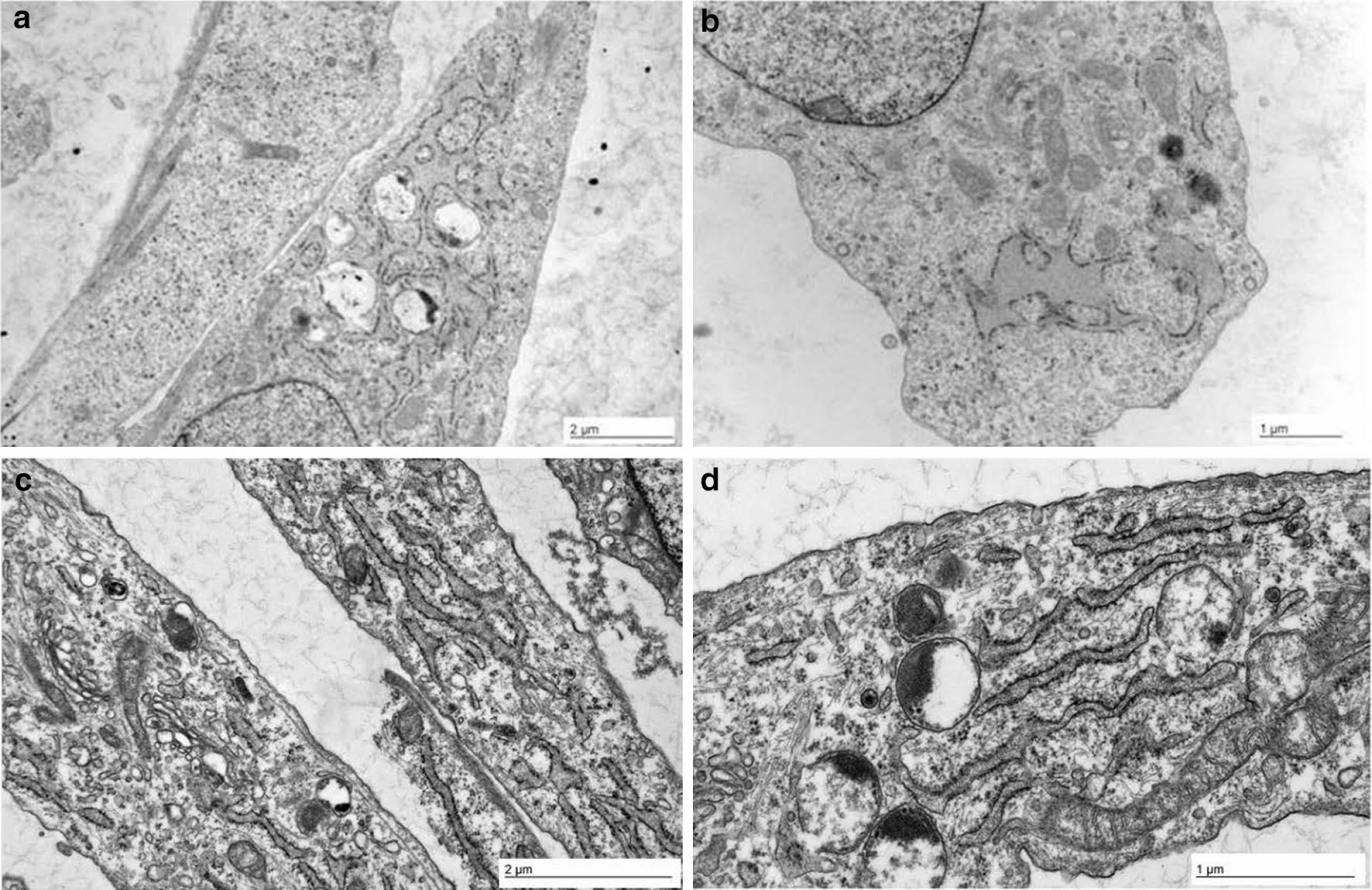
Transmission electron microscopy (TEM) analysis of cultured fibroblasts showing enlarged ER cisterns in the cells of the fetus (**a**, **b**) compared to a control (**c**, **d**)

The OI cells (Fig. 3a, b) presented with enlarged cisternae of the rough endoplasmic reticulum (rER) which were not seen in control fibroblasts (Fig. 3c, d).

## Discussion

Here we provide the first biochemical characterization of a mutation in the signal peptide of *COL1A1* leading to a severe form of OI and discuss possible underlying pathomechanisms.

Unfortunately, the availability of a single affected subject and the inaccessibility of osteoblasts present a limitation of our study. Despite this drawback, we show that it offers the opportunity to draw general conclusions on the pathomechanism involved in OI.

Firstly, we observed decreased collagen secretion, similar to the findings in OI with haploinsufficiency of *COL1A1*. However, haploinsufficiency is strongly associated with mild OI suggesting for additional pathological mechanisms responsible for the severe phenotype.

Furthermore, secretion of collagen I by the fibroblasts of the OI fetus was slightly delayed, but both collagen I chains showed a normal migration pattern suggesting that structural alteration of the ECM do not account for the severe phenotype in the fetus. This is in line with the fact that the mutation lies in a domain that is not part of the collagen fibrils in the ECM and also not involved in triple helical folding or processing. Unfortunately, bone tissue for mass spectrometry analysis was not available to definitively rule out the presence of aberrant collagen in the extracellular matrix.

Although the secreted type I collagen showed a normal migration pattern, a portion of collagen with aberrant modification appeared to be retained within the cell. The reduced secretion and aberrant modification of intracellularly retained collagen, not overlapping with the ER-luminal marker, suggest that the p.Gly22Arg mutation impairs cleavage and thus the release of preprocollagen α1(I) chains from the ER-entry sites (translocon). This is also supported by the results obtained by the in silico prediction of the cleavage efficiency. Impaired release of preprocollagen chains from the translocon might have pleiotropic effects, .e.g., leading to misfolding and ER stress or exhibiting a dominant negative effect on the COL1A1 chain encoded by the normal allele. Furthermore, blocking the translocon might affect cellular functioning since this pore is required for ER entry of other proteins of the secretory pathway, as well as for the export of misfolded proteins for ERAD or lysosomal degradation. Consistently, we detected dilated ER cisterns by electron microscopy, suggesting that ER stress might be involved in the pathology of OI as shown previously in a study analyzing a G610C mouse model of OI [15].

Pathological effects of signal peptide mutations have been characterized already in other genes (diseases) such as insulin (diabetes), *COL10A1* (Schmid-type metaphyseal chondrodysplasia, MCDS), and *COL5A1* (classical Ehlers–Danlos Syndrome, cEDS). These reports have shown that overall, mutations of the hydrophobic region cause protein deficiency as shown for COL5A1 in cEDS, whereas mutations of the cleavage site are associated with ER stress and dominant negative effects on the remaining wild-type protein as demonstrated for COL10A1 in MCDS and insulin in diabetes [16–18]. Thus, our findings are in line with previous reports on pathological effects of cleavage site mutations.

Taken together, our data support the hypothesis that intracellular mechanisms such as accumulation of unfolded proteins and ER stress can contribute to the pathophysiology of the more severe forms of OI (Sillence class II to IV). We suggest, therefore, that therapeutic approaches for OI aiming to increase the clearance of misfolded proteins should be taken seriously into consideration.
